# Dermatology

**Published:** 1905-06-10

**Authors:** 


### DERMATOLOGY.

**Adiposis Dolorosa.**—This curious and interesting condition was first described by Dercum in 1888, and is generally known in France as "Maladie de Dercum." About 50 cases have now been collected, and six autopsies have been made. Its ætiology, however, still remains obscure and its pathology doubtful, while no notable results have been established as to treatment. It may be regarded provisionally as a syndrome or symptomcomplex rather than a pathological entity, and clinically two forms may be distinguished—the nodular and the diffuse. The latter, which was the form first described by Dercum, and thought by him to resemble myxœdema is characterised by the deposition of loose fatty tissue on the lower extremities from the thigh to the ankle, the feet being free, subsequently the upper extremities to the wrists become involved, and the back and chest may also be invaded. The face and the abdomen are usually left unchanged. These deposits of fat are tender to touch and may be the seat of spontaneous pain. The patient is also afflicted with mental disturbances, there is marked asthenia, and the special senses are often affected. Trophic disturbances, such as loss of hair and patches of scleroderma are also noted. In the nodular form the fatty masses are more or less definite in outline, are tender, painful, and symmetrically distributed over the limbs, the hands, face, feet, and back being free. The disease is almost confined to the female sex, is very seldom hereditary, and has been known to follow various traumata. It has also occurred in subjects of alcoholism and syphilis, has followed ovariotomy, and in one case has been associated with polyuria. It is remarkable that a neurotic family history is seldom obtained. The most usually accepted view is that the condition is an intoxication, either endogenous or exogenous. In the former group, perverted secretion of one of the internal glands has been supposed. In the six autopsies, the thyroid was found diseased in four, and the pituitary body in two. The ovaries have also been thought responsible; in cases occurring before the menopause, that event has not taken place, and in some which occurred after it was established, irregular hæmorrhages from the uterus have recurred. The exogenous intoxicants are supposed to be those of alcohol, syphilis, tobacco, and possibly some special bacterial toxin. An interstitial neuritis of the finer nerves of the affected parts has often been demonstrated, and in two instances changes were found in the spinal cord, but these are now held to be secondary to the intoxication, however that may be produced. The large nerve trunks are apparently not affected. The diagnosis is seldom difficult, provided that the existence of the disease is borne in mind. The diffuse form was at first thought to resemble myxœdema, from which it may be distinguished by two points, apart from the absence of the spade hands and other characteristics of that disease. The age incidence is said to be higher in adiposis dolorosa, although cases in young women and even in children have been noted, and what is more important thyroid extract has little or no influence as a therapeutic measure. Elephantiasis and simple obesity are distinguished by the absence of mental symptoms, pain, and asthenia. The hereditary œdemas described by Meige and others have only a superficial resemblance, and occur moreover in families; the simple "segmental" œdema occurring over a portion of the lower extremities of young women, though possibly an allied condition, has obvious differences from true adiposis dolorosa. Reklinghausen's disease has been thought to resemble the nodular form, but in this condition the nodules are small and fibrous and occur along the distribution of nerve trunks. A curious condition described by Launois and Bensaude and called by them adenolipomatosis, consists of soft swellings on the nape and back, corresponding to certain lymphatic areas. The situation of the tumours, their occurrence mostly in males, and the fact that lymphoid tissue is found in them distinguish them from the multiple lipomata of Dercum. Two conditions are probably closely allied to adiposis dolorosa—namely, the multiple lipomata occurring in general paralytics and the neuro-arthritic elephantiasis described by Mathieu. In the latter there are swellings associated with joint pains and evidence of a polyneuritis. No treatment has been found to influence the disease to any extent. In one case surgical measures were successful, but in another recurrence took place almost immediately. No drugs have been uniformly useful. Good results have been reported from massage and electro-therapy in a few cases, but usually these methods are without marked effect.

